# Hooked embracing plate for Rolando fracture fixation: a preliminary result

**DOI:** 10.1186/s12893-022-01876-6

**Published:** 2022-12-08

**Authors:** Yueh-Ju Tsai, Jui-Po Yeh, Tsen-Hung Lin, Tsan-Shiun Lin, Ching-Hua Hsieh

**Affiliations:** Department of Plastic and Reconstructive Surgery, Kaohsiung Chang Gung Memorial Hospital, 123, Dapi Road, Niaosung Dist., Kaohsiung, 83301 Taiwan

**Keywords:** Rolando fracture, Thumb metacarpal base fracture, Hooked embracing plate, Hook plate

## Abstract

**Background:**

Rolando fracture is a comminuted, intra-articular fracture over the metacarpal bone base of the thumb which often leads to joint instability and requirement of surgery. The aim of this study is to evaluate the radiological and functional outcomes of Rolando fracture following surgical fixation with a hooked embracing plate (Acumed, 1.3 mm, Rolando Fracture Hooked Plate) designed for Rolando fracture.

**Method:**

We retrospectively reviewed a consequence of patients between 2018 and 2022 with Rolando fracture who received open reduction internal fixation with hooked embracing plates. Primary endpoints were the quality of radiologic reduction after the operation and peri-operative complications. Secondary outcomes were bone union, pinch and grip strength, palmar abduction, opposition and radiographic osteoarthritis over the trapeziometacarpal (TMC) joint.

**Results:**

A total of 5 patients were included. All patients had good quality of radiological reduction without peri-operative complications. The opposition, abduction, pinch and grip strength were nearly full-recovered for all patients with fine bone unions after 3 months follow-up.

**Conclusion:**

The hooked embracing plate is a good and safe option for surgical fixation in patients with Rolando fracture. Compared with traditional method such as lag screw or mini-plate fixation, the hooked embracing plate could provide rigid fixation with fine radiologic and functional outcomes with early mobilization.

## Introduction

Rolando fracture was firstly described by Silvio Rolando, an Italian surgeon, in 1910 [1]. It is classically a three-part, intra-articular fracture over the base of thumb metacarpal, which is described as “T” or “Y” morphologies [2]. The term has become to generally indicate intra-articular, comminuted fracture with 3 or more segments over the base of thumb metacarpal bone [3]. The injury is typically caused by compressive forces along the axis of the metacarpal bone while the trapeziometacarpal (TMC) joint in flexion [4]. The fracture would often lead to subluxation or instability of the TMC joint due to the tension force of the abductor pollicis longus (APL) tendon [5]. Surgical treatment is often needed due to its intrinsic instability [6].

The goal of treatment for Rolando fracture is to achieve anatomical reduction, perform stable fixation for early mobilization, optimize range of motion of the TMC joint, and to minimize pain [7, 8]. However, treatment is relatively difficult due to its intrinsic nature of instability and comminuted patterns, and complications as loss of reduction, joint incongruity or osteoarthritis could happen [4, 9].

The hook plate system was originated for mallet finger with small avulsed fragments [10]. Later, a hooked embracing plate (Acumed, 1.3 mm, Rolando Fracture Hooked Plate) was designed for intra-articular fracture over the base of the thumb metacarpal bone. However, few studies compare outcomes between the hook embracing plate and the traditional method (either lag screw or mini-plate) for patients with Rolando fracture receiving open reduction internal fixation. Therefore, we would like to share our experience on the hook embracing plate for Rolando fracture, and to evaluate the radiological and functional outcomes of our patients.

## Patients and methods

**Fig. 1:**
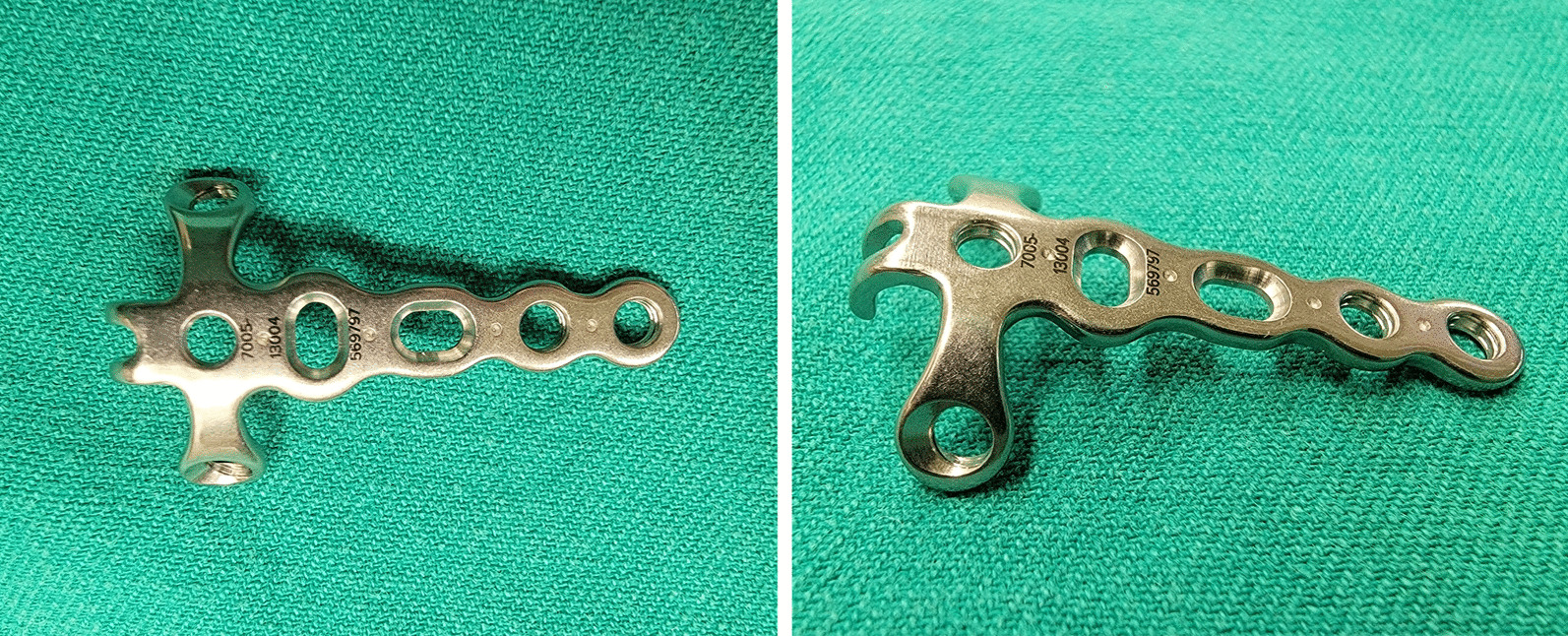
A hooked embracing plate (Acumed, 1.3 mm, Rolando Fracture Hooked Plate)

We retrospectively reviewed five patients with classic Rolando fracture or intra-articular comminuted fracture over base of thumb metacarpal bone from January 2018 to October 2022 in our hospital. All patients received open reduction internal fixation via a hooked embracing plate. The hooked embracing plate (Fig. 1) was produced by Acumed company as hand and wrist plating system. The study was approved by the Chang Gung Medical Foundation Institutional Review Board (CGMF-IRB, No. 202101329B0).

Patient demographics (age, sex, body mass index), underlying diseases, injury mechanisms and sites, fracture patterns, operations, and interval of mobilization were collected from medical records. The follow up was a minimum of 3 months. Primary outcomes were radiologic reduction after the operation and peri-operative complications including infection, neurovascular damage or loss of reduction. Secondary outcomes were bone union, palmar abduction, opposition according to Kapandji score [11], pinch and grip strength of the injured finger and radiographic osteoarthritis assessed by the Eaton-Littler classification [12].

## Surgical indication and technique

Hook embracing plate was recommended for patients with classic Rolando fracture and intra-articular, comminuted fracture over base of thumb metacarpal, just if the fracture bone segments were large enough for fixation. Patients with open fracture was excluded for the usage of the plate.

**Fig. 2:**
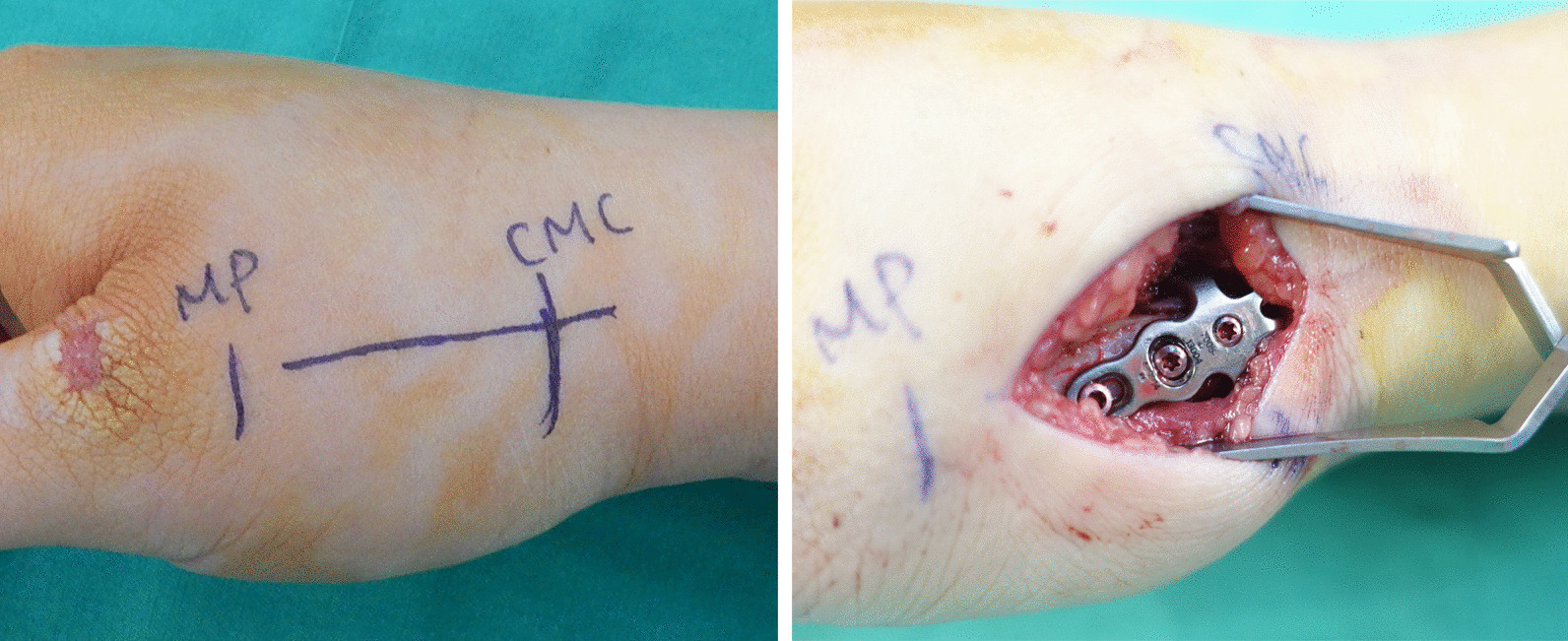
Surgical technique. (Left) Pre-operative marking of the metacarpophalangeal (MP) joint, the alignment of the metacarpal bone and the carpometacarpal (CMC) or trapeziometacarpal (TMC) joint. (Right) Intra-operative placement of and fixation of the hook embracing plate

All patients in our study received general anesthesia. A dorsal, straight incision was made over the base of thumb metacarpal bone [13] (Fig. 2). The sensory branch of radial nerve and radial artery was carefully protected during exploration of the wound [14]. The dissection went between the abductor pollicis longus and the extensor pollicis brevis tendon to disclose the metacarpal base [15]. The periosteum was opened with a sharp scalpel longitudinally and dissected carefully to exposed the fracture segments. Dissection would be stopped when getting close to the TMC joint to preserve the surrounding tissue of the joint capsule, such as connective tissue and ligamentous structure. We believed that this could also do less damage on the TMC joint leading to deterioration of joint instability.

The fracture segments were identified clearly, and to fragile pieces were removed. Traction force might help while doing the thumb reduction. Furthermore, stabilization of the basilar fracture segments was achieved with assistance of a temporary K-wire or reduction forceps [4, 9]. The 1.3 mm Rolando Fracture Hook Plate was then placed on the metacarpal bone for fixation. The hook on the proximal aspect of the plate was positioned onto the metacarpal base. A 2.3 mm lag screw would be inserted for inter-fragmentary fixation if it was applicable. The embraced arm portion of the plate was fixed to the basilar segments of the metacarpal bone with 1.5 mm screws. The metacarpal shaft was then fixed to the long portion of the hook plate with 1.5 mm screws. The periosteum was repaired to cover the hook plate. The skin was closed with Nylon sutures. A thumb spica splint was used for immobilization.

## Results

Five patients, including 3 males and 2 females, were documented with an average age of 40.8 years old (IQR 41.0 years old) (Table 1). All patients were injured due to a traffic accident. Four of the patients suffered from classic Rolando fractures with a three-part fragments, while the remaining one suffered from intra-articular, comminuted fracture over the base of thumb metacarpal bone. All fractures were close fracture. The average interval from injury to operation was 2.4 days (IQR 1.5 days). All patients underwent open reduction internal fixation with a hook embracing plate by dorsal approach. The average interval from operation to mobilization was 11.6 days (IQR 2.0 days). The average follow-up time was 8 months (IQR 12.0 months).

**Table 1:** Summary of cases

Case	Age/Sex	Injury mechanism	Fracture site	Open/close	Pattern	Approach	Operation	Mobilization interval (day)
1	56/M	TA, contusion	Right thumb, metacarpal base	Close	Rolando	Dorsal, straight incision	ORIF, hook plate	12
2	74/F	TA, contusion	Right thumb, metacarpal base	Close	Comminuted, intra-articular	Dorsal, straight incision	ORIF, hook plate	13
3	26/M	TA, contusion	Left thumb, metacarpal base	Close	Rolando	Dorsal, straight incision	ORIF, hook plate	12
4	26/F	TA, contusion	Right thumb, metacarpal base	Close	Rolando	Dorsal, straight incision	ORIF, hook plate	11
5	22/M	TA, contusion	Right thumb, metacarpal base	Close	Rolando	Dorsal, straight incision	ORIF, hook plate	10

M: male; F: female; TA: traffic accident; ORIF: open reduction internal fixation

All our patients achieved excellent radiologic reduction (Table 2). Joint incongruity was less than 1 mm in four of our patients, and around 1-2 mm in one of our patient. No joint subluxation happened. All patients had an acceptable angulation of less than 15 degree. No peri-operative complications including infection, neurovascular damage or loss of reduction were noted.

**Table 2:** Primary Outcomes

Case	Radiologic outcomes			Peri-operative complications		
	Joint incongruity	Subluxation	Angulation (degree)	Infection	Neurovascular damage	Loss of reduction
1	< 1 mm	(−)	< 15	(−)	(−)	(−)
2	1–2 mm	(−)	< 15	(−)	(−)	(−)
3	< 1 mm	(−)	< 15	(−)	(−)	(−)
4	< 1 mm	(−)	< 15	(−)	(−)	(−)
5	< 1 mm	(−)	< 15	(−)	(−)	(−)

Fine bone union was noted for all our patients after 3 months follow-up. All patients regained full opposition except one with a minimal disparity (Kapandji score 9/10) (Table 3). Abduction was good in four patients (> 45 degree) and fair in one case (30–45 degree). No significant difference in pinch and grip strength compared with the uninjured hand was noted except in one patient (less than 20%). Only one patient had a stage 1 Eaton-Littler radiographic osteoarthritis. All patients returned to their previous daily work and activities. Only one patient asked for removal of plate due to irritation after 20 months of surgery.

**Table 3:** Secondary outcomes

Case	Bone union (at 3 month)	Functional outcomes			Radiographic osteoarthritis (Eaton-Littler classification)
		Opposition (Kapandji score)	Abduction (degree)	Pinch and grip strength	
1	(+)	10/10	> 45	100	(−)
2	(+)	9/10	30–45	80	Stage 1
3	(+)	10/10	> 45	100	(−)
4	(+)	10/10	> 45	100	(−)
5	(+)	10/10	> 45	90	(−)

^*^ Pinch and grip strength, pinch and grip strength as the percentage of the contralateral values. OA, osteoarthritis

## Cases presentation

### Case 1

**Fig. 3:**
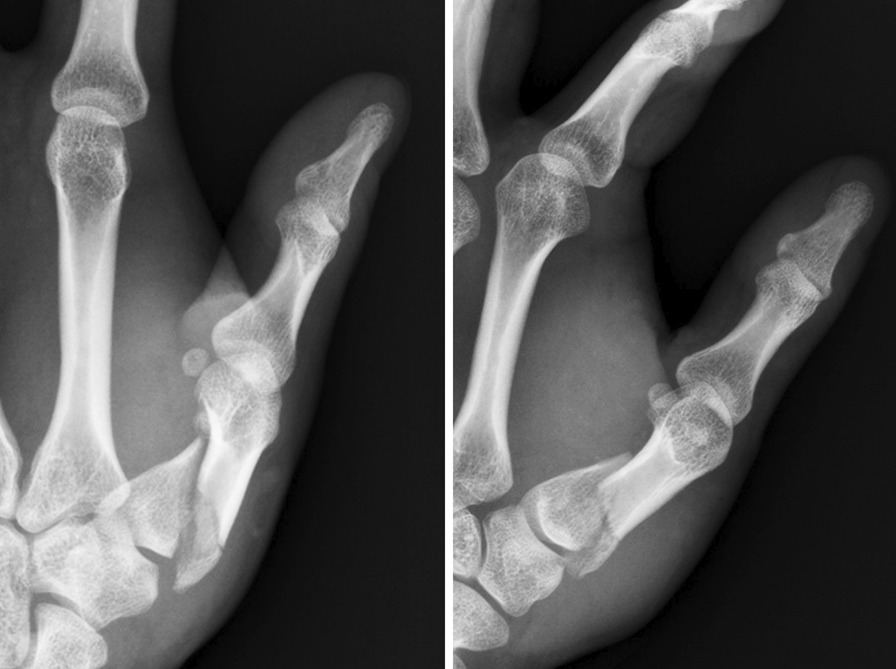
Left thumb metacarpal base Rolando fracture. (Left, A-P view) (Right, lateral view)

**Fig. 4:**
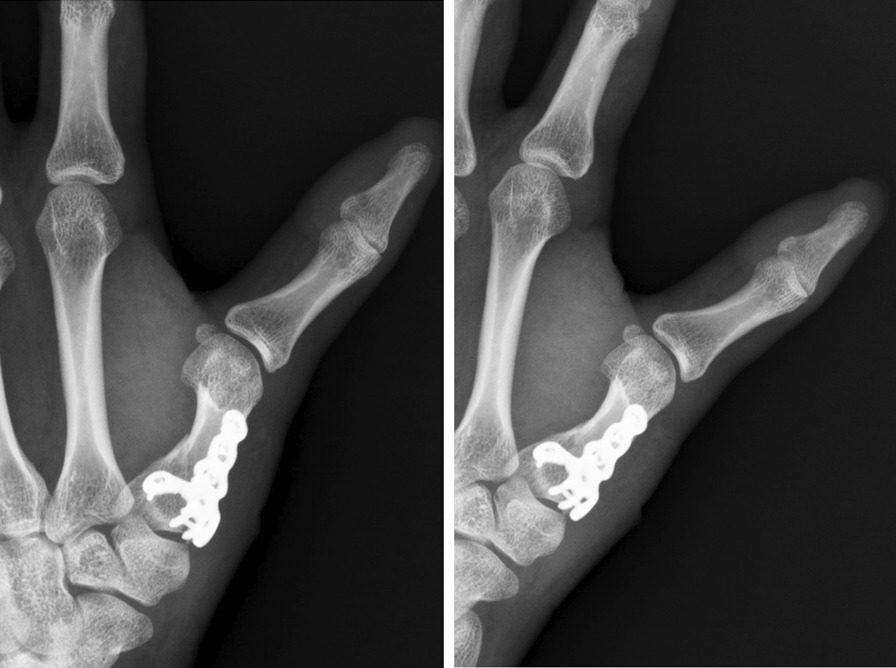
(POD 20) ORIF with a hook embracing plate with excellent radiologic reduction. (Left, A-P view) (Right, lateral view) * POD: post-operative day. ORIF: open reduction internal fixation

**Fig. 5:**
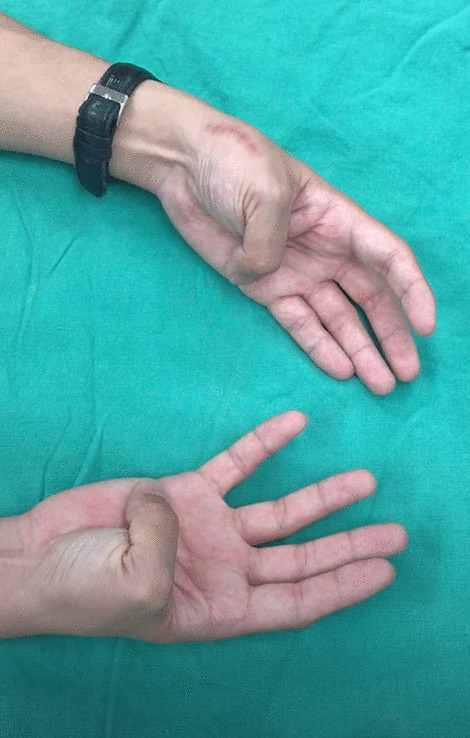
Fine functional outcomes with full opposition was noted over the injured hand after 5 weeks of surgery

A 26-year-old male patient (patient 3) was brought to the emergency department because of close, Rolando fracture over left hand due to contusion in a traffic accident (Fig. 3). He received open reduction internal fixation via dorsal approach with a hooked embracing plate 2 days after the injury. No peri-operative complications as infection or neurovascular damage was noted. The interval from operation to mobilization was 12 days. Radiologic reduction showed an excellent result after 20 days of surgery (Fig. 4). The left thumb achieved fine functional outcomes of range of motion after 5 weeks follow-up (Fig. 5).

### Case 2

**Fig. 6:**
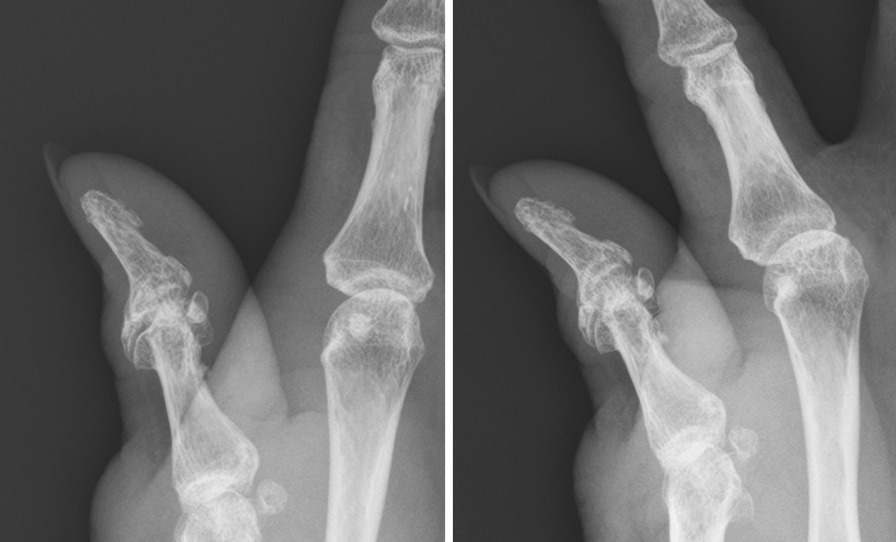
Right thumb metacarpal base Rolando fracture. (Left, A-P view) (Right, lateral view)

**Fig. 7:**
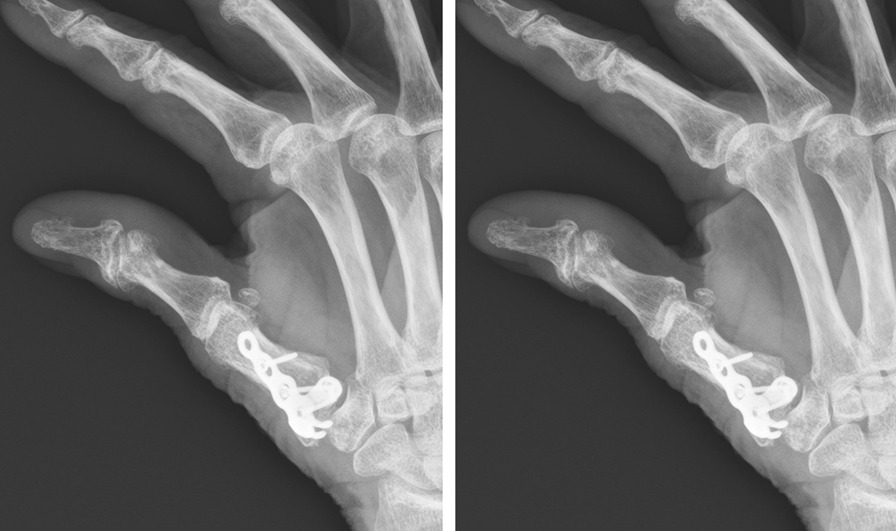
(POD 17) ORIF with a hook embracing plate with excellent radiologic reduction. (Left, A-P view) (Right, lateral view) * POD: post-operative day. ORIF: open reduction internal fixation

A 74-year-old female patient (patient 2) suffered from Rolando fracture over right hand due to a traffic accident (Fig. 6). She underwent open reduction internal fixation with a hooked embracing plate 3 days after the injury. There were no peri-operative complications nor neuromuscular damage after surgical intervention. The interval from operation to mobilization was 13 days. Radiologic reduction showed a good result after 17 days of surgery (Fig. 7). The opposition, fingers pinch, flexion and extension of right thumb were all good without disabilities after 4 months follow-up.

## Discussion

For Rolando fracture, conservative treatment with splinting seems to have poor reduction and functional outcomes in previous studies [15–17]. Surgical treatment is often needed due to its intrinsic instability [6].

Several studies revealed that close reduction percutaneous pinning (CRPP) might have unfavorable outcomes [16–19]. Van Niekerk et al. [17] reported 6 patients with Rolando fracture following CRPP. Most of the patients had poor radiologic reduction, prolonged casting period, limited opposition, and severe osteoarthritis after a 6-year follow up. Vichard et al. [16] also reported poor radiologic and functional outcomes of Rolando fracture following CRPP. On the other hand, Greeven et al. [18] revealed 3 patients with Rolando fracture undergoing CRPP with intermetacarpal K-wire with good functional outcomes over opposition of the thumb. However, two of the patients could not achieve stable fixation without an additional cast immobilization, and two of the patients had stage 1 Eaton-Littler radiographic osteoarthritis. One of the patient had loss > 30% grip strength compared with the contralateral side. Wang et al. [19] also reported 3 patients with Rolando fracture undergoing CRPP with intermetacarpal K-wire. They achieved fine radiologic and functional outcomes except limited abduction of thumb.

For Rolando fracture with large segments, open reduction internal fixation (ORIF) via either K-wire, lag screw or mini-plate seems to be preferable [2, 4, 6, 14, 20]. Levy et al. [21] reported 5 patients with Rolando fracture receiving open reduction internal fixation with K-wires or screws. The results showed good radiologic reduction and functional outcomes. However, they did not analyze the result of Rolando fracture from Bennett fracture individually, and they also excluded patients with comminuted fracture. Uludag et al. [8] revealed 7 patients with Rolando fracture undergoing ORIF with mini-plate or screw. All patients achieved fine bone union, anatomical reduction, early mobilization and full range of trapeziometacarpal joint motion, and had an acceptable grip and pinch strength loss < 20%. Mumtaz et al. [9] reported 9 patients with Rolando fracture having ORIF with mini-plate. Most of the patients had good functional results except one with pain, poor range of motion, and a stage 3 Eaton-Littler radiographic osteoarthritis. Four of the patients required removal of implant due to tenderness.

A hook embracing plate (Acumed, 1.3 mm, Rolando Fracture Hooked Plate) was introduced for Rolando fracture but with few evidence. In our study, all our patients achieved excellent radiologic reduction, fine bone union, good hand function and early mobilization after surgery with a hook embracing plate. Comparing with either K-wire, screw or mini-plate according to previous studies above [8, 9, 21], both the radiologic and functional results were non-inferior. No significant peri-operative complications including infection, neurovascular damage or loss of reduction were noted in our patients. Only one patient ask for removal of implant due to irritation after 20 months of surgery.

Restoration of articular surface is relative important [8, 9]. However, several studies did not found a strong relation between joint incongruity and osteoarthritis [2, 22]. Despite that, one of our patients with a post-operative joint incongruity around 1-2 mm suffered from a mild radiographic osteoarthritis without significant disability. Therefore, anatomical reduction should be achieved as possible to prevent such post-operative sequelae.

In our experience, the hook design allows an extra-bone-to-connective tissue support by positioned the hook on the ligamentous structure over the metacarpal base. The ligamentous structures around the TMC joint, especially the volar oblique ligament, play a big role in stabilization of the joint [4, 5, 15]. The extra-support could help further stabilize the fixation and diminish the joint deformity. Besides, the hook design allows us to restore the articular surface easier, preventing excessive bone stripping or joint capsule destruction in order to achieve anatomical reduction. Some studies mentioned that extensive dissection for open reduction could result in further damage of the hand [19, 20]. Therefore, by the usage of hook embracing plate, the periosteum and surrounding tissue could be persevered better, and it might be benefit on better circulation for bone union. Moreover, we thought the fixation might be more rigid from two dimensions of fixation screws by the design of embracing arm portion of the plate.

On the other hand, the price of the hook embracing plate was relative expensive than traditional lag screw or mini-plate, leading to the relative small case number in our studies. Besides, the technique is demanding and needs high degree of precision.

There were a few limitations in our study. First, it was a retrospective study of descriptive characteristic but no comparison with the results from other fixation methods or conservative treatment. Second, the included patient number was relatively small, and the follow-up time was relatively short. Most of the patients had limited pain or sequelae and were hard to ask coming back to outpatient department for a regular long-term follow up. Furthermore, few studies had evaluation of the surgical outcomes for Rolando fractures, and most of these studies had a small patient number. Therefore, meaningful comparisons of the outcomes with other studies became much more difficult.

Despite of the limitations, we still found that the hooked embracing plate provides fine preliminary result in patients with Rolando fracture, even in elder people with possible risks of osteoporosis. This method may provide more confidence of surgical fixation for post-operative early mobilization which also may contribute the better results. Therefore, open reduction via hooked embracing plate shall be considered as a better solution for Rolando fracture. Meanwhile, we can also design prospective studies with more detail comparison for further follow-up.

## Conclusion

The hooked embracing plate is a good and safe option for surgical fixation in patients with Rolando fracture. Compared with traditional method such as lag screw or mini-plate fixation, the hooked embracing plate could provide non-inferior radiologic and functional outcomes with fine bone union and earlier mobilization.

## Data Availability

All data generated or analyzed during this study are included in this published article.
